# Neurasthenia, from the Standpoint of the General Practitioner1Read before St. Louis Medical Society, January 20, 1894.

**Published:** 1894-04

**Authors:** I. N. Love

**Affiliations:** Professor Clinical Medicine and Diseases of Children, Marion-Sims College of Medicine, St. Louis; Vice-President American Medical Association; 3642 Lindell Boulevard


Buffalo Medical and Surgical Journal

Vol. XXXIII. APRIL, 1894. No. 9.

### Original Communications.

### NEURASTHENIA, FROM THE STANDPOINT OF THE GENERAL PRACTITIONER.[1]

By I. N. LOVE, M. D.,

Professor Clinical Medicine and Diseases of Children, Marion-Sims College of Medicine, St. Louis ; Vice-President American Medical Association.

SINCE Beard's time, the well-versed physician, whether in the general field or a special line, has recognized neurasthenia "as a legitimate and well circumscribed morbid entity." Our own Dr. C. H. Hughes, of St. Louis, recognized the world over as a high authority upon neurological subjects, in a paper read before the Missouri State Medical Association fully twelve years ago, suggested as a name for the disease, general functional neuratrophia, as preferable to neurasthenia. He expressed the thought at the time, that the disease was a more or less general failure of the normal nutrition appropriating power in the higher nerve centers, especially the psychical, leading to consequences short of appreciable structural change—a pure neuratrophia—which is only functional in its effects and confined, in expression, to an altered and lowered functionation in the nervous system itself.

A synonym for the disease is nervous prostration, and the name which Beard himself gave to it was nervous exhaustion, but all these names are objectionable, for they suggest symptoms rather than a definite pathological condition.

The causes of this disease, like all others, may be hereditary or acquired. The medical man who is brought in close relations to the family as family physician, should be fully impressed with the thought that his advice will be of value in the securing of nerve

1. Read before St. Louis Medical Society, January 20, 1894.

capital and a good general equipment for at least the rising generation of the family. Truly, many individuals are put into the world and have crowded upon them the battles of life when they are so poorly equipped with nerve force as to be almost considered nervous bankrupts.

The child which has a tubercular inheritance can be built up and away from it; so, too, if heredity is against it from the standpoint of the nervous system, the child may be fed up and out of it.

In these modern days of specialism, while admitting that the expansion of knowledge in every department of life has largely put an end to that ideal of "knowing something about everything, and everything about something," the members of the medical profession should yet guard themselves strenuously against the danger of automatic specialism; for, as was recently remarked by the *Medical Record*, "the glory of modern specialism is that it has unraveled difficult questions in the etiology of disease, and its crowning work must lie in its ability to meet the demands which this new pathological inquiry makes with adequate therapeutic measures. To do this, requires mental expansion in every direction, and not alone in any one."

As was well said by Dr. Hughes in his recent essay before this society upon this subject, "physicians may become specialists in practice, but they should never cease to be generalists as students."

The family physician, other things being equal, is well equipped for coping with the disease under consideration, within certain limits, on account of his personal knowledge of the habits, manner of life, and general physical make-up of the patient, as well as his temperament. Hutchinson defines temperament as the sum of the physical peculiarities of the man, exclusive of his tendency to disease, and it has tersely been expressed as being "the peculiar way in which the individual reacts to the stimuli of his environment." Temperament has been too much neglected in these latter days. There was a time, in professional and institutional circles, when much more stress was laid upon this subject, and an ability to recognize and know temperament used to be considered part of a sound medical training. There are many things in man which the test-tube and the microscope cannot discover, and in our work as bacteriological delvers, as well as in our desires to be superlatively scientific, we should not lose sight of this fact.

While by no means maintaining that leisure and wealth are the only conditions favorable to neurasthenia, so-called, I still urge, with Beard, that it is seldom found among those who live below the undercrust of the social world; that its *habitat* is rather in Fifth avenue than the Five Points. The manual laborers of the world, however little they may know, generally know enough to rest when they are tired, and they have the advantage of fewer superheated and poorly ventilated homes, besides their muscular development holds down their emotional centers to a safe level. Neither are they, as a rule, disturbed by the trinity of A's which confront a large proportion of those in the higher walks of life—namely, Ambition, Avarice and Anxiety, and the trio of L's which environ the rest—namely, Laziness, Luxury and Lust.

The writer desires to be placed upon record in favor of the position that, in spite of the fact that cases of neurasthenia are overlooked by the family physician, yet the number that are labeled nervous exhaustion by neurologists, which do not properly come under that head, is very greatly in excess; in other words, to a certain degree, it has become the fad on the part of the American public to elect to be placed under the head of neurasthenics, and neurasthenia is the chief hobby ridden by the nerve specialist of today. I take the position that ninety per cent. of the so-called cases of nervous exhaustion are spurious. That they are the victims of nervous weariness may be admitted, but weariness and exhaustion are two different things. Physical weariness may occur in three ways: the muscles may be affected, the nerves may become fatigued, the brain may become weary; all or any of the three may become tired out, and this tiring out may occur repeatedly time and again without exhaustion resulting; and the classical symptoms, as given by Beard and corroborated by so many other observers the world over, which apply to nerve exhaustion, may appear in a modified form in the victim of nerve weariness, and suggest to the alarmist, or he who is given to forming extreme conclusions, that the case before him is one of exhaustion.

Michael Foster, the great physiologist, in a recent address delivered before the members of the University of Cambridge, *apropos* to this subject of weariness, says:

Observations and reasonings, into the details of which I cannot enter now, have led physiologists to the conclusion that a muscle, not only in the body but also for a measurable time out of the body, is continually undergoing change of substance; that the complex groupings of atoms, molecules and particles, by virtue of which it is alive, are continually being made and as continually being unmade; the living complex muscle is always being built up out of, and always breaking down again into, simpler substances. Did we possess some optic aid which should overcome the grossness of our vision, so that we might watch the dance of atoms in this double process of making and unmaking in the living body, we should see the commonplace, lifeless things which are brought by the blood, and which we call food, caught up into and made part of the molecular whorls of the living muscle, linked together for a while in the intricate figures of the dance of life, giving and taking energy as they dance, and then we should see how, loosing hands, they slip back into the blood as dead, inert, used-up matter. In every tiny block of muscle there is a part which is really alive, there are parts which are becoming alive, there are parts which have been alive but are now dying or dead; there is an upward rush from the lifeless to the living, a downward rush from the living to the dead. This is always going on, whether the muscle be quiet and at rest or be moving; some of the capital of living material is always being spent, changed into dead waste; some of the new food is always being raised into living capital. But when the muscle is called upon to do work, when it is put into movement, the expenditure is quickened, there is a run upon the living capital, the greater, the more urgent the call for action. Moreover, under ordinary circumstances, the capital is, during the action, spent so quickly that it cannot be renewed at the same rate; the movement leaves the muscle with an impoverished capital of potential stuff, and a period of rest is indeed in order so that the dance of atoms of which I just now spoke may make good the loss of capital and restore the muscle to its former power.

In considering muscular weariness, we, at the same time, must keep in mind weariness of the nerve centers, for, after all, the weariness of the whole body from muscular work is, to a very great degree, in fact chiefly, a weariness, speaking broadly, of the brain.

When we have excessive muscular exertion, the weariness may take a form of distress, and, if the effort be continued, the distress may become so great as to cause complete exhaustion, and even death may result. In excessive work, of whatever kind it may be, in order for the work to be accomplished, there is made a greater demand upon the blood for oxygen. Difficult breathing, or panting, results from the changing quality of the blood. There are many things besides carbonic acid which are swept into the blood as the result of the activities of the body; in other words, the product of work in the human body is a poison which must needs be eliminated through the medium of the lungs and the other excretory organs.

Foster again well expresses it:

As the breath of man is poison to his fellow-man, so the outcome of the life of each part of the body, each tissue, be it muscle, brain, or what not, is a poison to that part and its fellows, and may be a poison to yet other parts. Of each member, while it may be said that the blood is the life thereof, it may with equal truth be said the blood is the death thereof; the blood is the channel for food, but it is also a pathway for poison.

It would seem superfluous to draw attention to these well-known physiological facts, but I do so only to emphasize the point which I make, and that is, that by neglect upon the part of the individual of certain necessary laws,—involving rest, opportunities for oxygenation, proper attention to elimination through the various draining channels of the body,—excessive work, whether bodily or mental, in repeated cases, produces such a degree of weariness and poisoning of the nerve centers as to mislead us in the direction of interpreting the symptoms present as those of neurasthenia.

The same author, in his admirable paper referred to, says:

The loss of living capital, or the presence of the products of work which would have no appreciable effect on a muscle, may wholly annul the work of a piece of nervous machinery. If an adequate stream of pure blood, of blood made pure by the efficient coöperation of organs of low degree, be necessary for the life of the muscle, in order that the working capital may be rapidly renewed and the harmful products rapidly washed away, equally true, perhaps even more true, is that of the brain. As physical and mental efforts are continued, the eliminating capacity, unless carefully guarded, is marred, the resulting poisons are more and more heaped up in the system, poison the muscles, poison the brain, poison the heart, poison, at last, the blood itself, starting in the intricate machinery of the body new poisons in addition to themselves. The hunted hare run to death dies, not because he is choked for want of breath, not because his heart stands still, its store of energy having given out, but because the poisoned blood poisons his brain, poisons his whole body. So, also, the schoolboy, urged by pride to go on running beyond the earliest symptoms of distress, the mere loss of wind, struggles on until the heaped up poison deadens the brain, and he falls dazed and giddy as in a fit, rising again it may be and stumbling on unconscious, or half conscious only, by mere mechanical inertia of his nervous system, on, only to fall once more, poisoned by poisons of his own making.

I quote liberally from the thoughts of the physiologist, Foster, for the purpose of making more clear the position taken. If continued effort, without rest, will poison to the extent of producing death, surely many repeated efforts which are not so much in excess with conditions favorable to accumulation of leucomaines, —namely, a tied-up state of the secretions, inattention to the proper diet, accumulations of undigested and unassimilated food, which undergoes fermentation and decomposition, developing ptomaines, thus adding to the reserve of poison in the system, keeping in mind the fact that the neglect of hygienic laws, failure to secure proper ventilation of rooms and the proper clearing out of the alimentary canal at proper times, surely we may have weariness and poisoning to the point of distress, and even to that point which would suggest nervous exhaustion. Men who are greatly absorbed in their work have no time to think about the laws of health, but the family physician, and the neurologist as well, should realize, in these cases, the importance of "clearing the decks for action." They should promptly turn on the batteries which will scatter the accumulated poisons, open the windows, put the patient out of doors, anywhere, so that the purest air may be breathed and elimination of the accumulated poisons hastened. In his essay, read before this society a few weeks ago, Dr. Bremer said:

There is no class of sufferers that is more persistently and vigorously maltreated than the neurasthenic. The cause of this lies in the fact that there is no organ, member or tissue, in the body, which has not been held up as the chief offender in neurasthenia by some or another specialist. The worst of it is, that since specialism is still on the increase, the number of offending parts of the body is steadily multiplying, and however much legitimate specialism may have done for the advancement of the medical sciences, it must be admitted that the abuse of specialism has assumed the proportions and significance of a nuisance. There is today no specialist who does not consider neurasthenia a legitimate and fruitful field for its work, in a few instances to the advantage, but much more frequently to the detriment of the patient. Broadly stated, the medical profession of today may be divided into two classes: the peripherists, comprising almost all specialists, and the centralists, found principally among the general practitioners and the neurologists. It has been said of the latter, that whenever they do make a right diagnosis, as a rule, nothing can be done for the patient. This is unfortunately true in a measure, but in this sense, that the patient has already gone the round of the peripherists, the specialists of the various organs, who mistook the case for being a local trouble, causing general symptoms, and tinkered at irrelevant symptoms, until the precious time for rational general therapeutics was gone. Had they looked upon the disorders of the various organs with a "central" light, interpreting the local trouble as being due to a general central disturbance, the result of their therapeutic efforts would probably have been entirely different.

As a matter of fact, it must be stated that social strata do not make any difference in the frequency of Beard's disease. Rich and poor are indiscriminately affected by it. The only difference is in name, not in fact. For while the well-to-do neurasthenic, especially he that has an inkling of the ailment from his reading on the subject, is apt to apply to a neurologist for relief, and get from him the true name of the disease, the poor man has to be satisfied with the diagnosis of biliousness, dyspepsia, catarrh and similar diagnostic incongruities, and is allowed to worry through his collapses as best he can, with the aid of quinine, calomel and tonics.

It is fortunate for humanity that the general pathologists and the other specialists of the world have had this note of warning given them by a neurologist. Possibly they will in future leave the domain referred to entirely to those who look upon all the disorders of the various organs with a "central" light and interpret all local troubles as being due to a general central disturbance. However positive this dictum may seem, practitioners and specialists will more than likely still go on recognizing the fact that over-eating, with too little defecation and accumulated leucomaines and ptomaines and peripheral irritation,—a figurative thorn in the flesh,—all may, if left unrelieved, create all of the symptoms of so-called neurasthenia, and that when relieved those symptoms will disappear as if by magic.

Commencing with the teething child which is thrown into reflex convulsions as the result of an erupting tooth, all along the line to the second childhood, when an unrelieved pruritis or eczema drives its victim mad with discomfort, we have evidences in favor of the thought that peripheral irritations are the prime cause in many cases of so-called neurasthenics. Every general worker and specialist in diseases of the rectum has found patients on the border line of nervous exhaustion and almost madness, all produced by a fissure of the anus. There are probably those within the sound of my voice who have seen numerous cases presenting many of the symptoms of neurasthenia, all occasioned by an obstructing spur upon one side or the other of the nasal septum, occasioning accumulations above, up to the frontal sinuses, and at times the sufferings approaching the agonies of the damned.

These cases, together with others which might be mentioned, the offending organs never having been interrogated, have run the gauntlet of neurological treatment for months and even years, and are finally relieved quickly and precisely by the removal of the peripheral disturbance. We all know that excruciating pain, borne even for a short time, or pain of any kind wherever located if long continued, will beget a general demoralization of the nervous centers, simulating nervous exhaustion. Surely all of us, whether specialists or workers in the general field, should try to save our patients pain, with a view to the husbandment of their nerve force. From infancy, the growing child should have its teeth guarded with a view to prevent toothache and the consequent nerve disturbance. The mothers of the world should be placed in the best possible condition for the pangs of maternity, remembering the element of poisoning, how susceptible the nervous centers are to being poisoned. Thus elimination, cleansing of all the emuncteries should be the rule, particularly during the pregnant period, and then every possible pain should be prevented. More rapid recoveries would follow and parturition would result in less loss of nerve force.

Indeed, there is no special line of study which the worker in general medicine or the family physician should apply himself to more assiduously than the nervous system. Just insofar as he can, this particular laborer in the medical vineyard should be a neurologist, for it is a fact, that all will admit, that after all the nervous system represents our main capital. All that will develop it and maintain it and prolong its usefulness, should be known and made a part of the armamentarium of the general practitioner. The neurological author of Current Fallacies about Neurasthenia, previously quoted, read a tirade in this society a few years ago against gynecology, (which, by the way, was never answered,) but may we not say that the criticism which may justly be directed toward the specialist who runs too much in grooves, will apply in a most pronounced way to the neurologist who dwells too much upon his particular line of thought?

In the language of the present president of this society, the brainy and brilliant Outten, "the psychiater, whose curious art is oft too finely wrought, syllogizes mind oft whilst his own is pathologized by thought." In other words, the neurologist is in danger of laying too great stress upon his particular part of the anatomy and is tempted to misinterpret symptoms and dislocate disease. To him, unless he have a care, every victim of suffering or discomfort is a bundle of nerves gone wrong. The most successful workers in this field are those whose emotional centers have been kept in reserve, and whose intellectual centers have been most strongly developed from the beginning, and who base their work upon a foundation of broad, general practice. The neurologist, like all the rest of us, should remember that every man becomes largely the crystallized result of all that he has seen, of that which he has associated with, and of that with which he has been brought most closely in contact. The psychiater, then, should constantly guard himself against exaggerating the neurological phenomena which present themselves to his view. He and the rest of us should remember that it is all very well to declaim against the specialist who interprets all headaches as being due to a necessity for eye-glasses, or the one who prefers to glide over and always only in gynecic grooves, but some of our neurological brethren, God bless them, have often been known to misinterpret and exaggerate symptoms. Admitting for the sake of argument that many a woman suffering from a sore brain or a sorrowing heart has received treatment for a sore womb, that the womb of the world has been twisted and twirled in excess, that the docile, gentle and inoffensive ovary has not often enough been permitted to blush unseen and waste its sweetness upon the silent air, but all too oft has been rudely snatched from its sacred lair when it never had been guilty of doing anybody harm, still the fact remains that gynecology has relieved more sorrows, brightened more homes, saved more precious lives, and will go on saving more millions of lives that exist now only in the womb of the future, than all of the other surgical specialties combined. It is the novices only who rush in where angels fear to tread and act upon the idea that a woman's anatomical, physiological and pathological world is her womb. We should none of us, however, throw stones at each other, for the bulk of those in the medical profession (and out of it too, for that matter) this side of Heaven, have their houses composed chiefly of glass.

I have in my records a half dozen cases of men of affairs approaching the fair, fat and forty age, where success has come to them after many weary years of labor; leisure, responsibility, overeating and constipation followed; then came headache, brain pressure, anxiety, insomnia, morbid fears followed by neurological opinions suggesting nervous exhaustion, which in its turn was followed by a frenzy of anxiety and dread of the future, all relieved within a few weeks by brisk purging, flushing of the kidneys, diaphoretics, fresh air, proper diet, a few nights of good sleep, and last, but not least, the instruction to buy and wear number sixteen collars instead of fourteen-and-a-half size which had been worn for years. Busy men when nearing the middle mile-post of life as they increase in weight, often fail to realize that their necks grow more rapidly than their girth, and, as a result, they suffer from plain every-day tight-collar headaches.

In a discussion on the subject at a recent meeting of this society, a prominent member cited his own case of neurasthenia as one produced by prolonged loss of sleep and anxiety on account of sickness in his household, and that he was practically cured by many long continued hours of sleep. Here was a case of extreme fatigue diagnosticated as nervous exhaustion, which was relieved by hours of sleep. Fancy, if you can anyone, the picture of intellectual health and animal vigor that he presents after having had neurasthenia and been cured of it inside of a few weeks.

Constipation, excessive eating, lack of exercise and absence of proper exposure to fresh air upon the part of one of the strongest, most healthy looking and most robust members of our profession, resulted in a collapse, which was interpreted as neurasthenia, treated as such, kept under treatment for several years, and the victim is not well yet, and the question may well be asked whether he ever will be. His neurologist flattered his vanity by telling him that he had neurasthenia as the result of overwork. Overwork forsooth! A practical all-around physician would have inquired into his habits of life and ascertained the fact that he was a gourmand, that he ate as much of the richest food, being possessed of epicurean tastes, as would have been sufficient for three men. Being a specialist, with his work confined almost entirely to ill-ventilated offices in an old styled, illy plumbed house on one of the prominent thoroughfares of the down-town portion of the city, the conditions were favorable to an accumulation of poisons. He was in the position of a furnace supplying force to a mechanism wherein the fuel was shoveled in in enormous quantities, a poor draft was given, the amount of oxygen was not sufficient, the ashes of combustion and clinkers were not properly removed, and as a result there was an explosion upon the part of the outfit. Had the doctor referred to fallen into the hands of an all-around many-sided physician, he would probably have been purged early and often for at least a week, for it would have taken that length of time, however energetic his doctor might have been, to have cleared out the accumulations in that alimentary canal. At the same time his kidneys would have been flushed with a view to the more complete removal of poisons which are eliminated through this channel. The activity of the skin would have been encouraged, and during this time the poisoned nerve centers having unfitted him for exercise, he would have been furnished muscular exercise through the medium of massage. I venture the opinion that he would have been rid of his leucomaine and ptomaine poisoning within a very short time, and having been placed upon a proper diet and instructed to take proper exercise, both in walking and horseback riding, he would have been in shape for work promptly.

Malaria, cold and rheumatic conditions are often potent factors of so-called cases of neurasthenia. Chilling of the surfaces, too, even if malaria be ruled out, and certainly where the system is below par, no matter what the case we may have under our care, malaria cannot often be ruled out of our philosophy, particularly in the Mississippi Valley. But even though it be not considered, with the nervous system (which, after all, is our power of resistance against disease) below par, the suspectibility to cold, so-called, is great. Chilling of the surfaces, driving the blood inwardly and interrupting the equilibrium of the circulation, still further adds to the difficulties which confront us, and begets a condition of disease which has been long considered under the term of rheumatic, to which Dr. Hans Froelich, in a paper recently presented to the profession, has given the name of lymphostasis.

Dr. Froelich says, "That with such patients there is always too rich a supply of food with too little combustion, caused by insufficient peristaltic and voluntary motion. These circumstances, single or combined, then, cause an engorgement of the concentrated lymph, which condition is first noticed in the fissures and tissue spaces, the source of the lymph system."

It will thus be seen that the rheumatic and gouty elements, the presence of uric acid and the deposit of fibromous materials along the track of nerves, in other words a lymphostasis, should be constantly kept in mind, and so too the fact that in proper regulation of diet, massage and flushing of eliminative organs, lies relief.

In closing, I desire to emphasize the following points:

1. The majority of cases of so-called neurasthenia are either victims of nerve weariness, dependent upon misdirected energy without proper rest at proper intervals; and nerve poisoning, the result of constipation, improper food and accumulation of the ashes of combustion, leucomaines and ptomaines; a disposition to use the stomach too much and the rectum too little, and a failure to appreciate the importance of pure air as a rejuvenator of tired and poisoned nature; or else nerve demoralization resulting from unrelieved peripheral irritations.

2. There is less harm in the victim of disease directing his attention to special organs and peripheral points as the offenders of his comfort, than there is in having his mind absorbed in the contemplation of his nervous apparatus. In other words, it is an evil day for anyone, man, woman or child, when the attention is concentrated upon the nerve centers, when the energies are directed toward watching nervous symptoms; in fact, it would be well for them if they never had a knowledge of the anatomy and functions of their nervous system, and that knowledge should be the last presented to them for consideration. The best way to cure the discomforts occasioned by nerve weariness and nerve poisoning, and even those of nervous exhaustion, which are far more serious, is to prevent them; and it should be our constant desire to impress upon the families with whom we come in contact the importance of building up the nerve capital of the child from the day of its birth, and even before its birth, indirectly through the mother. We should endeavor to teach the burden-bearers and battle-wagers of the world that the fruit-bearers have a nervous system more easily fatigued and more easily put on edge than their own. Then will we help them to realize that marriage is not a failure. With a view to the building up and maintainment of nerve force and guarding against nervous bankruptcy, both on the part of parents and children, we should teach them the importance of proper food, proper clothing and a properly opened condition of the animated system of sewerage. We should impress them with the fact that the daily visit to the Temple of Cloacus, with satifactory results, is quite as important as the morning and evening prayer. When we recall the fact that ninety per cent. of the women of the world are much more constipated than the traditional owl, we will realize what room there is for improvement in this direction. A special missionary work should be entered into, on the part of the profession, among the teachers of our schools, for they need not only to know the importance of the flushing out of the alimentary canal, so far as they themselves are concerned, but its importance as a stimulator of the wit and the ability of the young idea in the direction of shooting properly. All along the line we are safe in keeping in mind the cardinal principles, both as preventors and curers of the conditions mentioned,—namely, elimination, disinfection, nutrition, tranquilization and oxygenation. Large quantities of pure water serve, whether preceded by medicament or not, as excellent stimulators of the eliminative organs.

Food that is easily digested and readily assimilated is important, and, in this connection, the fats, hydrocarbons, are more valuable even as nerve builders than as enemies to tuberculosis, and that is saying a great deal. Under this head, butter and milk stand preëminent. Sleep is, indeed, "tired Nature's sweet restorer," and it represents the life of the individual. Mothers should be impressed with the fact that children, who are poor sleepers, run the chance of growing up with wrecked nervous systems. The average mother, as well as the average child, does not sleep enough.

The limits of this paper will permit of but few points in the way of treatment. As a sleep producer, I believe that trional in ten, twenty or thirty grain doses is the best remedy we have at hand. No exaltation, no depression, and no bad effects, follow its use. I observe, in a recent number of one of my exchanges, a very pronounced tribute to this remedy by Dr. J. B. Mattison, of Brooklyn, N. Y., a high authority. His experience is entirely in harmony with my own.

The digestion should be helped in order to secure nutrition in better form in every way possible. I have no sympathy with those who inveigh against the general aids to digestion. Just as well say that when a man's leg is broken or injured that he should not wear a crutch, as to say that when the digestive powers are crippled, crutches are not indicated. Pepsin, which assists in the digestion of albuminous foods, and diastase, which directs its energies toward the digestion of starchy foods, are of value. Papoid is also an excellent helper of digestion. Remembering that we have a condition of general debility all along the line, tonics are certainly indicated, and there is none better in all the world as a toner up of digestive and general nerve activity than strychnia.

For long, arsenic in the form of Fowler's solution has been a sheet anchor in the treatment of chorea and allied conditions, and believing, as I do, that in neurasthenia, which is a disease of adult life, the conditions are almost identical to those which are present during childhood when chorea prevails, (hysteria, chorea and neurasthenia are all a species of physical insanity,) I consider arsenic of great value. Within the past few months I have administered to these cases a preparation which was introduced to the medical profession by Dr. Wm. F. Barclay, of Pittsburg, (one of the most reputable physicians in the State of Pennsylvania, a man who is an expert chemist and a thoroughly reliable observer) and to which has been given the name of arsenauro, which is in fact a beautiful and attractive liquor of the bromide of gold and arsenic. (Every ten drops, the ordinary dose, contains one-thirty-second grain of gold bromide and one-thirty-second grain of arsenic bromide.) I have a very large number of patients taking this compound in ten-drop doses, three or four times a day and uniformly with good effect. Let us not forget that the majority of these cases, whether spurious or bona fide neurasthenia, have usually a rotten, crowded condition of the alimentary canal, a long history of constipation leading up to leucomaine and ptomaine poisoning, and that the entire system of secretory glands is deficient and perverted in activity, and that flushing out of the emunctory system is called for. As an all-around excellent stimulator of elimination, tongaline (liq. tong. salicylate-Mellier) is indicated. Pains which are present are well met by the salicylic acid contained in the compound and which is of the best form, being made from the oil of wintergreen. The pilocarpin, cimicifuga and colchicin which it contains are all stimulators of elimination. I usually administer from a teaspoonful to a tablespoonful at bed-time, and oftener if necessary in order to clear out the bowels thoroughly. In cases of la grippe, which are accompanied by so-called rheumatic symptoms and great nervous prostration, the tongaline is particularly indicated as a flusher of the sewerage system.

Change of scene and air are often indicated, and, to my mind, no better place in all America can present itself than Hot Springs, Arkansas. Here we have the elevated mountainous atmosphere, the ideal water, and facilities furnished for drinking it in large quantities, and being hot it is all the more promptly eliminated; and at the same time the bathing in the hot water is very beneficial in the direction of relieving the pains which so frequently accompany these conditions.

The judicious administration of electricity is certainly indicated, but I am strong in the belief that more of these cases are benefited by judicious massage, both manual and mechanical, than by electricity.

3642 LINDELL BOULEVARD.

